# Leg Ulcers in Sickle Cell Disease: A Multifactorial Analysis Highlights the Hemolytic Profile

**DOI:** 10.3390/hematolrep15010013

**Published:** 2023-02-15

**Authors:** Edvan do Carmo Santos, Paulo Vinícius Bispo Santana, Laíne Lopes Silva de Jesus, Gabriela Imbassahy Valentim Melo, Sètondji Cocou Modeste Alexandre Yahouédéhou, Caroline Conceição da Guarda, Rayra Pereira Santiago, Luciana Magalhães Fiuza, Suéllen Pinheiro Carvalho, Liz Oliveira dos Santos, Elisângela Vitória Adorno, Augusto Cezar Magalhães Aleluia, Luciene Cristina Gastalho Campos Luiz, Teresa Cristina Cardoso Fonseca, Marilda de Souza Gonçalves, Milena Magalhães Aleluia

**Affiliations:** 1 Laboratório de Patologia Aplicada e Genética, Departamento de Ciências Biológicas, Universidade Estadual de Santa Cruz, Ilhéus 45662-900, Brazil; 2 Laboratório de Investigação em Genética e Hematologia Translacional, Instituto Gonçalo Moniz, Fundação Oswaldo Cruz, Salvador 40296-710, Brazil; 3 Centro de Ciência e Tecnologia em Energia e Sustentabilidade, Universidade Federal do Recôncavo da Bahia, Feira de Santana 44042-280, Brazil; 4 Laboratório de Pesquisa em Anemias, Departamento de Análises Clínicas e Toxicológicas, Faculdade de Farmácia, Universidade Federal da Bahia, Salvador 40170-115, Brazil; 5 Departamento de Ciências Naturais, Universidade Estadual do Sudoeste da Bahia, Vitória da Conquista 45031-900, Brazil; 6 Departamento de Ciências da Saúde, Universidade Estadual de Santa Cruz, Ilhéus 45662-900, Brazil

**Keywords:** leg ulcers, sickle cell disease, hemolysis

## Abstract

Sickle cell disease (SCD) is characterized by the presence of the variant S hemoglobin (HbS). The homozygous genotype (HbSS) is sickle cell anemia (SCA), while the double heterozygous of HbS and HbC (HbSC) is defined as SC hemoglobinopathy. The pathophysiology is based on chronic hemolysis, inflammation, endothelial dysfunction, and vaso-occlusion, which results in vasculopathy and serious clinical manifestations. Sickle leg ulcers (SLUs) are cutaneous lesions around the malleoli frequent in 20% of Brazilian patients with SCD. SLUs present a variable clinical and laboratory pattern modulated by several characteristics that are not fully understood. Hence, this study aimed to investigate laboratory biomarkers and genetic and clinical parameters associated with the development of SLUs. This descriptive cross-sectional study included 69 SCD patients, 52 without SLU (SLU−) and 17 with active or previous SLU history (SLU+). The results showed a higher incidence of SLU in SCA patients and there was no observed association of α-3.7 Kb thalassemia in SLU occurrence. Alterations in NO metabolism and hemolysis were associated with clinical evolution and severity of SLU, in addition to hemolysis modulating the etiology and recurrence of SLU. Our multifactorial analyses demonstrate and extend the role of hemolysis driving the pathophysiological mechanism of SLU.

## 1. Introduction

Sickle cell disease (SCD) is a group of hemoglobinopathies characterized by the presence of the variant S hemoglobin (HbS). The homozygous form (HbSS) is defined as sickle cell anemia (SCA) [1,2,3]. HbS can also be inherited with the variant hemoglobin C (HbC), which leads to hemoglobinopathy SC (HbSC) [4,5,6]. SCD is associated with several clinical manifestations, such as pulmonary hypertension, stroke, vaso-occlusion, priapism, sickle leg ulcers (SLUs), and other systemic manifestations [7].

SLUs are cutaneous lesions that frequently affect the malleoli in lower extremities [8,9,10]. Usually, SLUs are superficial lesions with elevated edges and may present bloody, serous, or purulent exudate [11]. The SLU wound bed may present unviable tissues, such as necrotic tissue, owing to the accumulation of dead cells or sloughy tissue, which is characterized by cell fragments and absence of vascularization. Furthermore, the SLU wound bed may also have viable tissues, such as granulation tissue, which corresponds to angiogenesis, as well as epithelized tissue, consisting of regenerated and dry epidermis [8,11]. In addition, the size of the SLU, the period of time in which the SLU remained open, and the SLU recurrence are important characteristics whose influencing factors are not fully understood [12].

In this sense, intravascular hemolysis, sterile inflammation, reduced nitric oxide (NO) bioavailability, and endothelial dysfunction have been associated with SCD pathophysiology [13]. However, this association is not fully explained in SLU occurrence nor is its role in variable clinical evolution of SLU.

Genetic parameters are usually insufficiently explored in studies involving SLUs. Genetic modifiers, such as alpha deletion of 3.7 kb thalassemia (α-3.7 Kb thalassemia), may be useful in elucidating the pathophysiology in SLUs [14]. Likewise, individuals with SCD who carry α-3.7 Kb thalassemia may present laboratory alterations related to increased blood viscosity, such as a higher red blood cell (RBC) count, hemoglobin (Hb), and hematocrit (Hct) concentration, in addition to lower mean corpuscular volume (MCV), mean corpuscular hemoglobin (MCH), and mean corpuscular hemoglobin concentration (MCHC) [15].

Hence, considering the association between hemolysis and SLU, and the importance of understanding the multifactorial mechanism associated with SLU development, the present study aimed to characterize the SLU. We investigated laboratory biomarkers, genetics, and clinical parameters associated with SLUs.

## 2. Materials and Methods

### 2.1. Study Design

The present descriptive cross-sectional study included 69 SCD patients, 42% (29/69) with SCA and 58% (40/69) with HbSC disease, all seen at the Itabuna Sickle Cell Disease Reference Center (CERDOFI), in the state of Bahia, Brazil. Seventy-five percent (52/69) patients did not present SLU (SLU−) and 25% (17/69) presented active SLU or reported a previous history (SLU+). Regarding hydroxyurea (HU), 82.3% (14/17) of SLU+ patients and 53.8% (28/52) of SLU− patients were taking HU; owing to clinical severity, these patients could not stop using HU at the time of the study. All patients that were in steady state were included in the study, and this condition was defined as an absence of painful vaso-occlusive crises and absence of blood transfusion for 3–4 months. Patients without SCD (HbSS and HbSC) were excluded from the study. This work was conducted according to the guidelines of the Declaration of Helsinki and approved by the Institutional Review Board of the Santa Cruz State University (UESC) (protocol number: 11765319.7.0000.5526). Informed consent was obtained from all subjects involved in the study.

### 2.2. Clinical Data

The clinical data were collected using a standardized and confidential questionnaire self-reported at the time of the study enrollment and later confirmed by the medical records at CERDOFI. The clinical manifestations presented by the patients in the last six months of the day of the blood collection were investigated. Among SLU+ patients, 76.5% (13/17) had active SLU and 23.5% (4/17) had healed SLU at study enrollment. The clinical descriptions of active SLUs were performed by the medical and nursing staff of CERDOFI and recorded in the medical record.

### 2.3. Laboratory Determinations

Laboratory analyses were performed at Genetics and Applied Pathology Laboratory (LAPAGEN), UESC, as well as at Clinical Analyses Laboratory (LACTFAR), College of Pharmaceutical Sciences, Federal University of Bahia (UFBA). The nitric oxide metabolites (NOm) and genetics analyses were conducted at the Laboratory of Investigation in Genetics and Translational Hematology (LIGHT), Gonçalo Moniz Institute (IGM), Oswaldo Cruz Foundation (FIOCRUZ), Bahia, Brazil.

Hematological analyses were performed using an automatic hematology analyzer ABX Pentra 80 (HORIBA Medical, Montpellier, France). Reticulocytes were counted after staining supravitally with brilliant cresyl blue dye. Hemoglobin profiling and determination of HbF levels were assessed by high-performance liquid chromatography using an HPLC/Variant II hemoglobin testing system (Bio-Rad, Hercules, CA, USA).

Serum levels of lactate dehydrogenase (LDH), total bilirubin and fractions, aspartate aminotransferase (AST), alanine aminotransferase (ALT), gamma-glutamyl transferase (GGT), urea, creatinine, uric acid, and iron were assessed using an automatized equipment CB 400i (Wiener Lab, Rosario, Argentina). Ferritin levels were determined using an Architect i1000SR (Abbott, Chicago, IL, USA).

The NOm were determined in serum samples using Griess reagent, according to the method previously described [16].

To perform genetics analyses, leukocyte genomic DNA was extracted employing the QIAamp extraction kit (Qiagen, Hilden, Germany). Allele-specific PCR was used to detect the α-3.7 Kb thalassemia deletion, according to the method previously described [17].

### 2.4. Statistical Analyses

Statistical analyses were conducted using the software program Statistical Package for the Social Sciences (SPSS) version 22.0 (IBM Software, New York, NY, USA). GraphPad Prism version 6.0 (Graphpad Software, La Jolla, CA, USA) was used for graph assembly. Shapiro–Wilk test was used to determine quantitative variables’ distribution. The Mann–Whitney U test and independent *t*-test were used to compare the groups according to the normality of the distribution for each variable. Linear regression multivariate (LRM) analysis was performed to evaluate possible associations between the recurrence of SLUs and laboratory biomarkers with regard to the outcome of interest, SLU. The principal component analysis (PCA) is a common method employed for the identification of predictor factors in medical sciences [18]. A 15 × 9 matrix was assembled and data were previously auto-scaled in order to diminish the differences between the magnitudes. PCAs were performed using Statistica 10.0 software (StatSoft Inc., Tulsa, OK, USA). *p* values < 0.05 were considered statistically significant.

## 3. Results

### 3.1. Clinical Characterization of SLUs

The results showed that 41.2% SLU+ patients experienced the first SLU during pediatric age (13–17 years), while 35.2% experienced it during adulthood (18–30 years) (Table 1). The leading cause of SLU onset was trauma in the lower extremities (Table 1).

Additionally, 64.7% SLU+ patients presented SLU in the left or right malleolus, while 23.5% presented SLU on both malleoli simultaneously (Table 1). Further, 17.6% SLU+ patients developed SLUs that were open for 3 months and 82.4% for 4 months or years (Table 1). Moreover, 82.4% SLU+ patients presented up to three SLU recurrences, along with 17.6% who presented four to seven SLU recurrences (Table 1).

Clinical characterization of active SLUs in 13 SLU+ patients showed that 100% of the wound bed consisted of sloughy tissue; 53.8% was necrotic tissue; 46.1% was granulation tissue; and 7.6% was epithelized tissue. Serous (100%) and purulent (53.8%) exudate were prominent. Additionally, 46.1% of the SLU presented integrate edges; 38.4% were dehydrated and 30.7% presented tissue maceration (Table 1). In addition, hyperpigmented tissue was present in 77% of the perilesional skin, dry tissue was present in 38.4%, and swelling was present in 38.4% (Table 1).

### 3.2. Laboratory Parameters in SLU− and SLU+ Patients

The results showed that SLU+ patients had lower RBC, segmented neutrophils and absolute reticulocyte counts, and Hb and Hct levels, as well as higher HbS, HbF, MCV, MCH, and platelet counts (*p* < 0.05) (Table 2).

In addition, SLU+ patients had higher LDH, total and indirect bilirubin, AST, GGT, uric acid, and iron levels (*p* < 0.05) (Table 3).

### 3.3. Genetic Parameters in SLU− and SLU+ Patients

Among SCA and HbSC patients, 76.5% of SLU+ patients were SCA and 23.5% were HbSC (*p* < 0.05). Among SCA patients, the mean age of the first SLU was 20.9 ± 8.4 years, whereas in HbSC patients, the mean age was 34.0 ± 14.0 years (*p* < 0.05).

In the group of SCD patients without α-3.7 Kb thalassemia, 75% were SLU− patients and 25% were SLU+ patients, in addition to 80% SLU− patients and 20% SLU+ patients among patients with α-3.7 Kb thalassemia (*p* > 0.05) (Table S1).

Additionally, laboratory biomarkers were associated with α-3.7 Kb thalassemia among SLU− and SLU+ patients. Regarding patients without α-3.7 Kb thalassemia, SLU+ patients had a lower RBC count and Hb and Hct concentration, in addition to higher MCV, MCH, LDH, total and indirect bilirubin, AST, GGT, and iron levels (*p* < 0.05) (Table S1). Among patients with α-3.7 Kb thalassemia, SLU+ patients had a lower RBC count, Hb, Hct, and MCHC concentration, in addition to higher MCV, MCH, LDH, total and indirect bilirubin, AST, and iron levels (*p* < 0.05) (Table S1).

### 3.4. Hemolytic Index in SLU Occurrence

**Figure 1:**
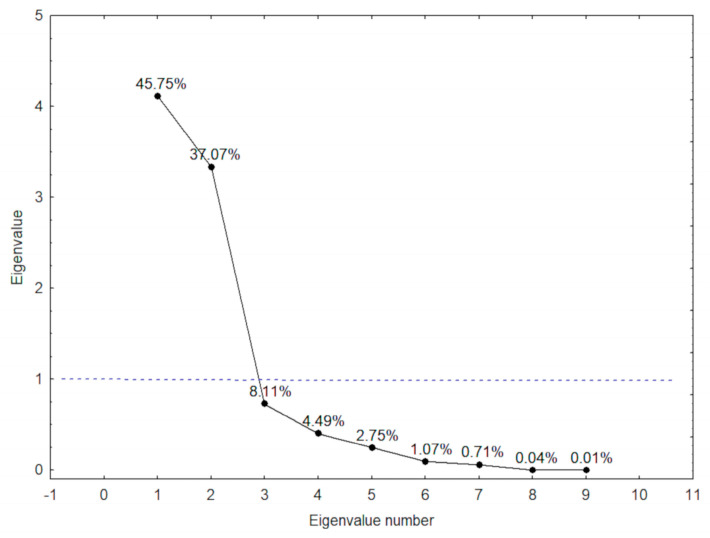
Significance assessment among laboratory biomarkers in patients with sickle leg ulcers or previous history (SLU+) obtained by eigenvalues to Principal Component Analysis (PCA) results.

PCA was performed to identify predictive factors for SLU+ patients. The results showed two principal components (PCs) with 82.8% of total variance (Figure 1).

The two PCs were hemolytic biomarkers that are strongly correlated with SLU occurrence, as the coefficients were higher than 0.5 (Table 4).

**Figure 2:**
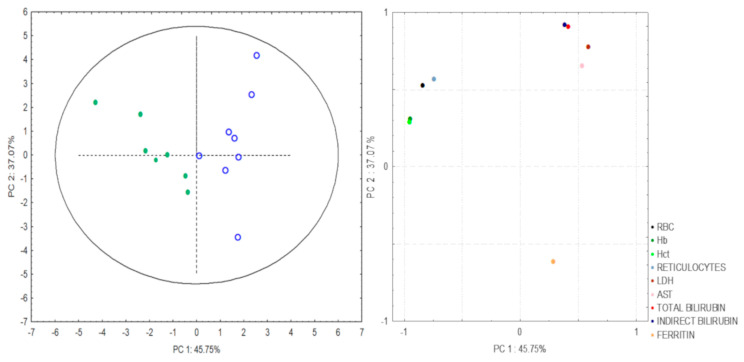
Scores of the samples and loading graph obtained by PC1 and PC2 to determine the association between hemolytic biomarkers and sickle leg ulcers. PC, Principal component; RBC, red blood cells; Hb, hemoglobin; Hct, hematocrit; LDH, lactate dehydrogenase; AST, aspartate aminotransferase.

Correlation analyses between PCs and different variables demonstrated that PC1 was responsible for 45.75% of the total variance, including RBC and reticulocyte counts, as well as Hct and Hb levels. PC2 was responsible for 37.07% of total variance, including LDH, AST, total and indirect bilirubin, and ferritin levels (Figure 2).

### 3.5. Hemolytic Biomarkers and HbF Levels in SLU Recurrence

A linear regression multivariate (LRM) model was performed with SLU recurrence as the dependent variable. Our model shows that Hb, Hct, LDH, indirect bilirubin, ferritin, and HbF levels were independently associated with SLU recurrence (Table 5).

### 3.6. Laboratory Biomarkers and Clinical Characteristics of SLU

**Figure 3:**
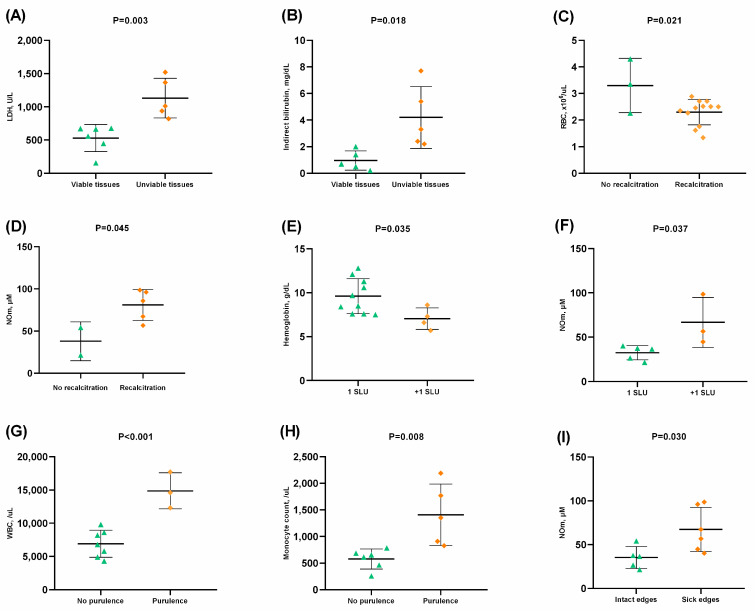
Associations between laboratory biomarkers and clinical characteristics of active sickle leg ulcers (SLU). (**A**) Higher lactate dehydrogenase (LDH) levels in SLU with unviable tissues; (**B**) Higher indirect bilirubin levels in SLU with unviable tissues; (**C**) Lower red blood cell (RBC) count in SLU with recalcitration (>6 months); (**D**) Higher nitric oxide metabolites (NOm) levels in SLU with recalcitration (>6 months); (**E**) Lower hemoglobin concentration in patients with more than one SLU; (**F**) Higher nitric oxide metabolites (NOm) levels in patients with more than one SLU; (**G**) Higher white blood cell (WBC) count in SLU with purulence; (**H**) Higher monocyte count in SLU with purulence; (**I**) Higher nitric oxide metabolites (NOm) levels in patients with sick edges in SLU. *p*-value obtained using independent *t*-test.

The SLU consisted of unviable tissues, especially sloughy and necrotic tissues, presenting increased LDH (Figure 3A), indirect bilirubin (Figure 3B), total bilirubin, HbS, and AST levels (*p* < 0.05). Interestingly, SLUs with unviable tissues were identified exclusively in SCA patients (*p* < 0.05).

SLU+ patients with recalcitrant SLU (>6 months) presented decreased RBC counts (Figure 3C) and Hb levels, as well as increased NOm (Figure 3D), HbS, and HbF levels (*p* < 0.05). SLU+ patients who had more than one SLU also presented lower Hb (Figure 3E) and Hct, as well as higher NOm (Figure 3F), AST, and ferritin levels (*p* < 0.05).

Serous and purulent exudates were associated with increased white blood cells (WBC) (Figure 3G) and monocyte (Figure 3H) and lymphocyte (*p* < 0.05) counts, as well as HbS, ferritin, and uric acid levels (*p* < 0.05). Sick edges were associated with increased NOm levels (*p* < 0.05) (Figure 3I).

## 4. Discussion

The underlying mechanism responsible for SLUs is thought to be multifactorial. In our study group, the first SLU occurred after 12 years of age, which is in agreement with previous studies [13,19,20]. Most of the SLU+ patients presented lesions in the lateral or medial malleolus, and a few patients had lesions in both malleoli. Malleolar involvement may be justified by thin skin, a low amount of local subcutaneous fat, in addition to the expressive susceptibility of the marginal microcirculation to be obstructed by sickled RBCs, generating low blood flow and intravascular erythrocyte destruction [9,21].

Trauma in the malleolus has been suggested as the primary physical risk factor for SLU opening [8,19]. We observed that most SLU+ patients experienced traumatic episodes in malleoli, such as scratches, insect bites, and domestic accidents, prior to SLU opening [19,22].

Investigation of laboratory parameters showed higher levels of HbS, LDH, AST, and total and indirect bilirubin in SLU+ patients. These results reinforce the previous notion that SLUs are associated with SCD hemolytic subphenotype [19,23,24]. In addition, reduced RBC count and decreased Hb and Hct concentration in SLU+ patients correspond to anemia. This hemolysis promotes endothelial dysfunction and vasculopathy, while local inflammation, characterized by intense production of pro-inflammatory cytokines and leukocytes, such as neutrophils and monocytes, enhances the ongoing pathophysiology [13,25,26]. The PCA demonstrated two hemolytic components associated with SLUs. Thus, this paper corroborates previous studies that demonstrate the association between hemolysis markers with different clinical manifestations in SCD [27,28].

Additionally, SLUs were more frequent in SCA patients compared with HbSC; likewise, SLUs occurred earlier in SCA patients than in HbSC patients. Therefore, individuals who carry the HbSS genotype, the most severe genotype of SCD, may be at a greater risk of developing SLUs [13]. Regarding α-3.7 Kb thalassemia, there was a low frequency in SLU+ patients; however, the hemolytic profile in the SCD was not influenced by the deletion [29]. Interestingly, SLU+ patients showed a 23.5% increase in uric acid levels in comparison with SLU− patients. Previous studies suggest that increased uric acid levels may indicate erythrocyte hyperplasia in the bone marrow in response to hemolysis [25]. It may also indicate increased oxidative stress, resulting in reduced NO bioavailability [25,30]. These findings reinforce the association of high uric acid levels with hemolysis in SLU occurrence.

Hemoglobin metabolism in SCD is characterized by elevated indirect bilirubin levels and subsequent production of gallstones, which could cause cholelithiasis and increased GGT levels, as a hemolysis consequence [31]. In this study, SLU+ patients presented higher GGT levels. Ballas (1991) observed that SCD patients presented SLU and cholelithiasis simultaneously, although no association was found [32]. In our study, none of the SLU+ patients reported a diagnosis of cholelithiasis. However, high levels of GGT and indirect bilirubin reinforce the chronic hemolysis associated with SLU occurrence and predisposition to cholelithiasis.

Previous evidence about HbF in SLUs is controversial as there is no consensus about a protective role [12,19,33]. This study found higher levels of HbF in SLU+ patients, while an LRM model demonstrated that HbF levels were independently associated with SLU recurrence (R^2^ = 0.981; *p* < 0.05). Despite these observations, this study was unable to determine the influence of HbF in SLUs because 60.8% (42/69) of SCD patients were taking HU. HU is the main pharmacological therapy for SCD, known for increasing HbF concentrations [25].

Regarding SLU recurrence, a study proposed that frequent hemolysis added to microvascular injury may be the main risk factor for inefficient healing and SLU reopening [8]. The LRM model from this study found that hemolytic biomarkers were independently associated with SLU recurrence for up to seven episodes (R^2^ = 0.981; *p* < 0.05). Therefore, the results suggest that chronic hemolysis is associated with the predisposition to SLU recurrence and its variability.

Laboratory biomarkers were also associated with clinical aspects of SLUs. We found hemolytic biomarkers in association with necrotic and sloughy tissues in SLUs, which is in agreement with hemolytic anemia. Previous analysis indicates that hemolysis and anemia are predictive factors that may result in unsuccessful treatment of SLUs [12]. SLUs remained open from weeks to years among SLU+ patients, which demonstrates the variability in healing time. SLU+ patients with recalcitrant SLUs (>6 months) had severe anemia. Additionally, patients with more than one SLU simultaneously had altered hemolytic biomarkers levels. Therefore, SLUs with unviable tissues, recalcitration, and multiple SLU occurrence might be modulated by hemolysis that favors vasculopathy and delays healing. Furthermore, SLUs present predominantly purulent exudates and association with inflammatory cells, suggesting active inflammation and increased susceptibility to local clinical severity.

Additionally, NOm levels were associated with recalcitrant SLUs, simultaneous SLU occurrence, and sick edges in SLUs, which suggests that alterations in NO metabolism lead the severity of clinical characteristics in SLUs.

## 5. Conclusions

In summary, the results of this study corroborate the SLU pathophysiology as multifactorial because laboratory biomarkers and genetic and clinical parameters are responsible for modulating the SLU etiology, clinical evolution, and recurrence. The descriptive systematization of data associating laboratory biomarkers related to hemolysis with clinical aspects of SLUs has been rarely performed. Thus, we believe our results promote the understanding of both local and systemic alterations that may be useful to improve clinical practice.

## Figures and Tables

**Table 1:** Clinical characterization of SLU.

**Leg ulcer age at onset (years)**	**N (%)**	**Leg ulcer location**	**N (%)**	**Origin**	**N (%)**
13 to 17	7 (41.2)	Right malleolus	6 (35.3)	Traumatic	10 (58.8)
18 to 30	6 (35.2)	Left malleolus	5 (29.4)	Spontaneous	7 (41.2)
31 to 46	4 (23.6)	Right and left malleolus	4 (23.5)		
		Atypical	2 (11.8)		
**Leg ulcer duration (months)**	**N (%)**	**Leg ulcer recurrence (episodes)**	**N (%)**	Wound bed tissue *	**N (%)**
1 to 3	3 (17.6)	1 to 3	14 (82.4)	Necrotic tissue	7 (53.8)
4 to 12	4 (23.5)	4 to 7	3 (17.6)	Sloughy tissue	13 (100)
13 to 24	4 (23.5)			Granulation tissue	6 (46.1)
25 to 36	2 (11.9)			Epithelial tissue	1 (7.6)
>37	4 (23.5)				
Exudate characteristics *	**N (%)**	Lesion edges *	**N (%)**	Perilesional skin *	**N (%)**
Serous	13 (100)	Maceration	4 (30.7)	Hyperpigmentation	10 (77.0)
Bloody	1 (7.6)	Dehydration	5 (38.4)	Dryness	5 (38.4)
Purulence	7 (53.8)	Healthy	6 (46.1)	Edema	5 (38.4)
				Healthy	3 (23.0)

SLU, sickle leg ulcers. * only 13 patients with active sickle leg ulcer.

**Table 2:** Hematological characterization of SLU− and SLU+ patients.

Laboratory Parameters	SLU− (N = 51)	SLU+ (N = 17)	*p*-Value
	Mean ± SD	Mean ± SD	
**Hb pattern**			
HbS, %	62.61 ± 17.52	76.55 ± 15.73	**0.005**
HbF, %	4.46 ± 5.70	9.52 ± 7.96	**0.024**
**Erythrocytes**			
RBC, ×10^6^/μL	3.71 ± 1.03	2.62 ± 0.92	**<0.001**
Hemoglobin, g/dL	10.59 ± 2.89	9.19 ± 2.51	0.037 *
Hematocrit, %	32.21 ± 7.36	27.75 ± 7.38	**0.010**
MCV, fL	92.20 ± 13.43	109.07 ± 17.08	**<0.001**
MCH, pg	30.47 ± 4.64	36.07 ± 5.75	**<0.001**
MCHC, g/dL	33.04 ± 0.94	33.07 ± 0.75	0.896
RDW, %	17.53 ± 2.76	17.25 ± 3.05	0.733
Reticulocyte count, %	3.57 ± 1.41	2.95 ± 1.22	0.110
Reticulocyte count, /mL	137,078 ± 72,073	81,537 ± 51,981	**0.005**
**Leukocytes**			
WBC, /μL	9226 ± 3369.9	8458 ± 3786.8	0.433
Segment count, /μL	4639 ± 2329.42	3184 ± 1621.44	**0.020**
Eosinophil count, /μL	352 ± 441.83	327 ± 337.30	0.832
Basophils count, /μL	28.24 ± 55.58	24.24 ± 50.75	0.794
Lymphocyte count, /μL	3375 ± 1394.17	4021 ± 2094.30	0.249
Monocyte count, /μL	831 ± 372.78	901 ± 519.47	0.546
**Platelets**			
Platelet count, ×10^3^/μL	331.55 ± 137.34	426.12 ± 184.54	**0.028**
MPV, fL	9.87 ± 1.23	9.41 ± 0.99	0.165

SLU, sickle leg ulcers; SLU−, patients without sickle leg ulcers; SLU+, patients with active or previous history of sickle leg ulcers; Hb, hemoglobin; HbS, hemoglobin S; HbF, fetal hemoglobin; RBC, red blood cell; MCV, mean cell volume; MCH, mean corpuscular hemoglobin; MCHC, mean corpuscular hemoglobin concentration; RDW, red cell distribution width; WBC, white blood cell; MPV, mean platelet volume. *p*-value obtained using independent *t*-test. * *p*-value obtained using Mann-Whitney *U* test. Bold values indicate significance at *p* < 0.05.

**Table 3:** Biochemical characterization of SLU− and SLU+ patients.

Laboratory Parameters	SLU− (N = 48)	SLU+ (N = 16)	*p*-Value
	Mean ± SD	Mean ± SD	
**Hemolysis markers**			
LDH, U/L	579.33 ± 326.14	763.19 ± 347.95	0.021 *
Total bilirubin, mg/dL	1.73 ± 2.07	2.88 ± 2.20	0.024 *
Direct bilirubin, mg/dL	0.39 ± 0.91	0.31 ± 0.16	0.119 *
Indirect bilirubin, mg/dL	1.33 ± 1.41	2.56 ± 2.16	0.022 *
**Hepatic profile**			
AST, U/L	34.56 ± 28.95	55.81 ± 39.88	**0.025**
ALT, U/L	22.60 ± 15.54	23.56 ± 16.10	0.833
GGT, U/L	46.59 ± 33.11	88.19 ± 67.71	**0.030**
**Renal profile**			
Urea, mg/dL	23.63 ± 9.23	26.38 ± 19.57	0.450
Creatinine, mg/dL	0.75 ± 0.19	0.78 ± 0.38	0.757
Uric acid, mg/dL	5.22 ± 1.84	6.45 ± 2.68	**0.044**
**Iron metabolism**			
Iron, mcg/dL	103.58 ± 43.059	146.81 ± 68.059	**0.027**
Ferritin, ng/mL	411.74 ± 488.91	867.39 ± 2360.13	0.455

SLU, sickle leg ulcers; SLU−, patients without sickle leg ulcers; SLU+, patients with active or previous history of sickle leg ulcers; LDH, lactate dehydrogenase; AST, aspartate aminotransferase; ALT, alanine aminotransferase; GGT, gamma-glutamyl transferase. *p*-value obtained using independent *t*-test. * *p*-value obtained using Mann-Whitney U test. Bold values indicate significance at *p* < 0.05.

**Table 4:** Laboratory parameters associated with SLU determined by PCA.

PC	Variables	r
**1**	RBC	**−0.84**
	Hb	**−0.95**
	Hct	**−0.96**
	Reticulocytes	**−0.74**
**2**	LDH	**0.71**
	AST	**0.59**
	Total Bilirubin	**0.84**
	Indirect Bilirubin	**0.85**
	Ferritin	**−0.68**

SLU, sickle leg ulcers; PCA, principal component analysis; PC, principal component; r, correlation; RBC, red blood cell; Hb, hemoglobin; Hct, hematocrit; LDH, lactate dehydrogenase; AST, aspartate aminotransferase.

**Table 5:** Linear regression multivariate model of SLU recurrence in aassociation with laboratory biomarkers.

Independent Variables	Dependent Variable	*p*-Value	β	R^2^	*p*-Value of the Model
RBC, ×10^6^/mL	**Recurrence of SLU**	0.226	0.647	**0.981**	**0.013**
Hemoglobin, g/dL		**0.004**	10.691		
Hematocrit, %		**0.002**	−12.284		
LDH, U/L		**0.005**	1.216		
Indirect bilirubin, mg/dL		**0.002**	−2.270		
Ferritin, ng/dL		**0.014**	−0.746		
HbF, %		**0.011**	−0.911		

SLU, sickle leg ulcers; RBC, red blood cell; LDH, lactate dehydrogenase; HbF, fetal hemoglobin; R^2^: coefficient of determination; β: coefficient of regression; Bold values indicate significance at *p* < 0.05. Recurrence of SLU was defined as up to seven episodes of reopening.

## Data Availability

Data are owned and saved by the affiliated CERDOFI and are available upon request to the corresponding author. For researchers meeting the criteria for access to confidential data, please contact the following email address: mmaleluia@uesc.br.

## Supplementary Materials

The following supporting information can be downloaded at https://www.mdpi.com/article/10.3390/hematolrep15010013/s1, Table S1: Laboratory parameters of SLU− and SLU+ patients according to α-3.7 Kb thalassemia.
