# Supplementary material for: Leg Ulcers in Sickle Cell Disease: A Multifactorial Analysis Highlights the Hemolytic Profile
Source: Hematol Rep. 2023 Feb 15;15(1):119–29. doi: 10.3390/hematolrep15010013 (PMC9945128; doi:10.3390/hematolrep15010013)
Supplement: Supplementary file 1 [file hematolrep-15-00013-s001.zip › hematolrep-1994364-supplementary.pdf]

Table S1: Laboratory parameters of SLU- and SLU+ patients according to $\alpha^{-3.7Kb}$ thalassemia.

	**αα/αα, n = 52**			**-α/αα and -α/-α, n = 15**		
**Hematological biomarkers**	**SLU- (n=39)**	**SLU+ (n=13)**	***P-value***	**SLU- (n=12)**	**SLU+ (n=3)**	***P-value***
RBC, x10⁶/μL	3.79 ± 1.03	2.66 ± 1.05	**0.002***	3.04 ± 1.07	2.39 ± 0.36	**0.043***
Hemoglobin, g/dL	11.0 ± 2.85	9.35 ± 2.64	**0.049***	10.3 ± 1.49	7.80 ± 1.80	**0.036***
Hematocrit, %	33.9 ± 7.46	28.1 ± 7.79	**0.021***	32.1 ± 5.11	24.0 ± 5.32	**0.034***
MCV, fL	92.3 ± 14.4	110 ± 17.0	**0.001**	91.4 ± 10.9	113 ± 5.51	**0.022***
MCH, pg	30.5 ± 4.98	36.5 ± 5.67	**0.001**	30.1 ± 3.75	36.9 ± 1.55	**0.022***
MCHC, g/dL	33.0 ± 1.00	33.2 ± 0.78	0.655	33.2 ± 0.41	32.4 ± 0.35	**0.031***
RDW, %	17.3 ± 2.85	17.0 ± 3.19	0.750	18.3 ± 2.51	18.5 ± 3.21	0.908
**Hemolytic biomarkers**						
LDH, U/L	544 ± 280	784 ± 387	**0.025**	518 ± 143	813 ± 277	**0.046**
Total bilirubin, mg/dL	1.30 ± 1.16	2.88 ± 2.27	**0.039**	1.63 ± 0.83	3.60 ± 2.15	**0.028**
Direct bilirubin, mg/dL	0.25 ± 0.30	0.33 ± 018	0.388	0.27 ± 0.09	0.30 ± 0.10	0.742
Indirect bilirubin, mg/dL	1.05 ± 0.96	2.55 ± 2.24	**0.045**	1.33 ± 0.79	3.30 ± 2.10	**0.024**
AST, U/L	27.4 ± 17.4	53.9 ± 35.1	**0.026**	35.1 ± 19.5	95.0 ± 74.9	**0.035**
GGT, U/L	43.8 ± 27.3	84.3 ± 63.5	**0.019***	38.7 ± 21.5	100 ± 93.3	0.064
Uric acid, mg/dL	5.38 ± 1.88	6.62 ± 2.84	0.092	4.79 ± 1.79	6.45 ± 2.19	0.252*
Iron, mcg/dL	97.5 ± 38.7	138 ± 46.8	**0.005***	121 ± 52.7	265 ± 82.0	**0.005**
Ferritin, ng/mL	319 ± 337	298 ± 289	0.842	311 ± 222	296 ± 392	0.936
**Nitric oxide metabolism**						
NOm, μM	49.2 ± 20.4	52.2 ± 23.5	0.762*	46.2 ± 20.1	27.6 ± 3.16	0.070*

SLU-, patients without sickle leg ulcers; SLU+, patients with active sickle leg ulcers or previous history; RBC, red blood cell; MCV, mean cell volume; MCH, mean corpuscular hemoglobin; MCHC, mean corpuscular hemoglobin concentration; RDW, red cell distribution width; LDH, lactate dehydrogenase; AST, aspartate aminotransferase; GGT, gamma-glutamyl transferase; NOm, nitric oxide metabolites. *P*-value obtained using independent t-test. **P*-value obtained using Mann-Whitney *U* test. Bold values indicate significance at $P < 0.05$.
